# Methodological Considerations for Preterm Birth Research

**DOI:** 10.3389/fgwh.2021.821064

**Published:** 2022-01-11

**Authors:** Thokozile R. Malaba, Marie-Louise Newell, Landon Myer, Vundli Ramokolo

**Affiliations:** ^1^Division of Epidemiology and Biostatistics, School of Public Health and Family Medicine, University of Cape Town, Cape Town, South Africa; ^2^School of Human Development and Health, University of Southampton, Southampton, United Kingdom; ^3^School of Public Health, University of the Witwatersrand, Johannesburg, South Africa; ^4^Centre for Infectious Disease Epidemiology and Research, School of Public Health and Family Medicine, University of Cape Town, Cape Town, South Africa; ^5^HIV Prevention Research Unit, South African Medical Research Council, Cape Town, South Africa; ^6^Gertrude H. Sergievsky Center, Vagelos College of Physicians and Surgeons, Columbia University Irving Medical Center, New York, NY, United States

**Keywords:** preterm birth (PTB), low middle income countries (LMICs), bias (epidemiology), methodology, maternal infections, HIV, antiretroviral therapy (ART)

## Abstract

Complications from preterm birth are a leading cause of infant mortality, with long-term implications for morbidity and quality of life of preterm infants. There are many important risk factors for preterm births however in this article, we focus on the maternal infection etiological pathway, given its significance in low-to-middle income countries. In high preterm birth settings such as sub-Saharan Africa, maternal HIV infection and antiretroviral therapy (ART) use have been associated with an increased risk of preterm births. Consequently, we highlight methodological considerations related to selection and measurement bias in preterm birth research. We further illustrate the potential impact of these biases in studies investigating the relationship between HIV/ART and preterm births. We also briefly discuss issues related to population-level estimations based on routinely collected clinical or civil registration data. We conclude by emphasizing the importance of strengthening of antenatal care services to improve quality of population data as well as optimizing current and future study designs, by taking into account the important methodological considerations described in this article.

## Introduction

Preterm birth (PTB) is defined by the duration of gestation at the time of delivery with a cut-off of 37 weeks distinguishing preterm from term infants. As the leading cause of neonatal and child mortality and its impact on child development it has possible long-term implications for quality of life. An estimated 14.8 million PTB occur globally each year (1), however they are not equally distributed. Low-to-middle income countries (LMICs) have a higher burden than other countries; in particular sub-Saharan Africa and South Asia which account for 60-80% of global PTB (1, 2). Addressing PTB in LMICs is therefore essential for accelerating progress toward achieving Sustainable Development Goal (SDG) 3 “Ensuring healthy lives and wellbeing for all at all ages” partly by ending preventable deaths of newborns and children under five by 2030 (3). Continued focus on improving the survival and quality of life of these infants born too soon is needed—with an emphasis on optimizing appropriate evidence-based PTB prevention and mitigation interventions in LMICs. High-quality epidemiologic data are necessary to determine current and emergent modifiable risk factors during the preconception, pregnancy and peripartum period. This is best achieved through well-designed studies which take into account important methodological considerations. In this article we briefly discuss some of these considerations.

## Epidemiologic Study of Preterm Birth in LMICS

Preterm birth is a complex syndrome with varying phenotypes and multifactorial etiologies, each with distinct biological pathways. In addition to gestational age classification, PTB can also be classified phenotypically according to clinical presentation into spontaneous or medically-indicated PTB (4). At the individual level accurate PTB identification is important for appropriate clinical management; while at the population level it is crucial for informing policy formulation and resource allocation (5).

The epidemiologic study of PTB requires robust methods of quantifying population-level estimates, through national civil registration and vital statistics (CRVS) systems that report PTB (1). However, many LMICs still have inadequate health information systems and sub-standard statistical capacity (6). This is demonstrated by the fact that the regions with the highest PTB burden, sub-Saharan Africa and South Asia, contributed little to no CRVS data in the latest global PTB estimates (1). Most data used to estimate PTB in these settings is not nationally representative. Instead, the data come from surveys (e.g., Demographic and Health Surveys), poorly designed small-scale research studies and/or secondary and tertiary care facilities which cater to specific subsets of the total population. Consequently, in these settings, high-quality real-time data from prospective studies and secondary analyses of robust existing data are critical. This is particularly important given the increased use of novel artificial intelligence techniques like machine learning (ML). The best ML models use high quality real time and existing data for predictive modeling and early diagnosis of health outcomes such as PTB (7). Unlike traditional statistical models, ML can handle more complex data structures. Furthermore their ability to incorporate different types of data (e.g., laboratory tests, imaging, and clinical notes) can provide better outcome phenotyping (8).

Pathways to spontaneous PTB include decidual hemorrhage, maternal/fetal stress, hypothalamic-pituitary-adrenal activation, pathologic uterine overdistension or cervical insufficiency and infection/inflammation, the most common pathway in LMICs (9, 10). Accordingly, understanding the overall contribution of infections (and their treatment) to PTB is essential. Given that PTB is a common outcome, small increases in risk can have substantial public health impact in settings where neonatal care services are limited. This is particularly important in sub-Saharan Africa, the epicenter of the HIV infection pandemic (11) and where HIV is one of the leading complications of pregnancy. It should be noted that antiretroviral therapy (ART) for HIV treatment, while essential for improving maternal health and survival, has also been implicated in increased PTB risk (12–14).

## Methodological Considerations

The practical and ethical costs of conducting randomized trials in pregnant women necessitates the use of observational study designs, which are subject to a variety of potential biases (15). When considering study design, data collection and analytical procedures for these studies, taking selection and information bias into account is critical. Preterm birth analyses are further subject to bias because they are not usually the primary objective of the study but are rather planned or unplanned secondary analyses. Consequently data collection procedures are often not designed to examine important aspects of PTB, so the best high-quality measures and procedures are not always utilized. To strengthen the validity of associations drawn from these observational studies of PTB, it is important to identify and evaluate potential sources of bias—especially those common and unique to perinatal research.

### Maternal Antiretroviral Therapy Use and Preterm Birth: Methodological Example

In LMICs numerous studies have investigated the infection pathway to PTB, given its contribution to PTB in these settings. As an example, we draw on the vast body of literature on the relationship between HIV/ART and preterm birth in high HIV burden settings. We also highlight some methodological nuances that underlie these epidemiologic investigations. It should be noted that these concepts can be extended to other infections and/or exposures.

The relationship between maternal ART use and PTB has been an area of research for many years, with conflicting results from multiple studies. HIV infection has been shown to increase the risk of PTB through poor maternal health, increased risk factors for coinfections and fetal HIV infection (16). ART use introduces complexity to deciphering associations with PTB and other adverse birth outcomes, because it improves maternal health and reduces acute retroviral fetal infection. It would seem logical that healthier women have better birth outcomes; however, ART could also increase adverse birth outcomes through other mechanisms. This complicates the understanding of these competing forces and makes the epidemiology of this challenging.

**Figure 1:**
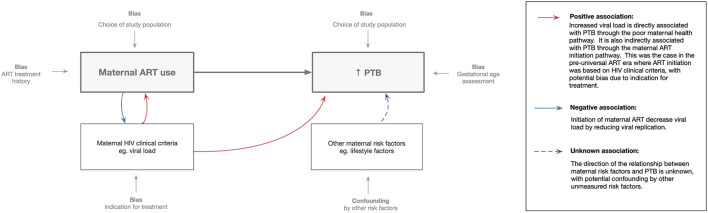
Methodological perspective: association between maternal ART use and preterm birth. Maternal ART use assessment is influenced by treatment history (including timing of initiation and ART regimens used) and study population chosen. Preterm birth assessment is influenced by the accuracy of gestational age measurement and the study population (i.e., trimester of inclusion of women in the study).

In studies of among women receiving ART in pregnancy, some have suggested increased risk of PTB while others have found no evidence of associations with PTB. Putative explanations for these inconsistent findings have been linked to differences in study designs, study populations, HIV treatment guidelines and analytical approaches. The exposure (maternal ART use) and outcome (PTB) are both significantly impacted by selection and measurement bias (Figure 1).

Using examples of recent studies, we highlight instances where selection and measurement bias impacted study findings.

#### Selection Bias

This is the distortion of an association due to the omission or inclusion of specific groups of women, such that the sample no longer reflects the population of interest (17). Perinatal study populations are dynamic and complex because the reproductive process spans from fertilization and implantation to clinically recognized pregnancy, and further to birth and early childhood. Processes of selection (e.g., implantation failure, early pregnancy losses) and attrition (e.g., stillbirths, neonatal deaths) render these populations incompletely observable (18, 19). Studies investigating outcomes of pregnancy are subject to further selection, because recruitment is often based on a convenient sample of women willing and able to access routine care services early in pregnancy. Given that early antenatal care initiation is sub-optimal in LMICs (20), the women recruited likely represent those with better health seeking behaviors. During enrolment, additional selection can occur if women do not meet study specific eligibility criteria. This is often related to gestational age and/or the absence of an exposure or outcome of interest. Clinical interventions delivered during pregnancy (e.g., elective terminations, activity restriction or induction) can also directly influence PTB incidence and impact study findings.

**Figure 2:**
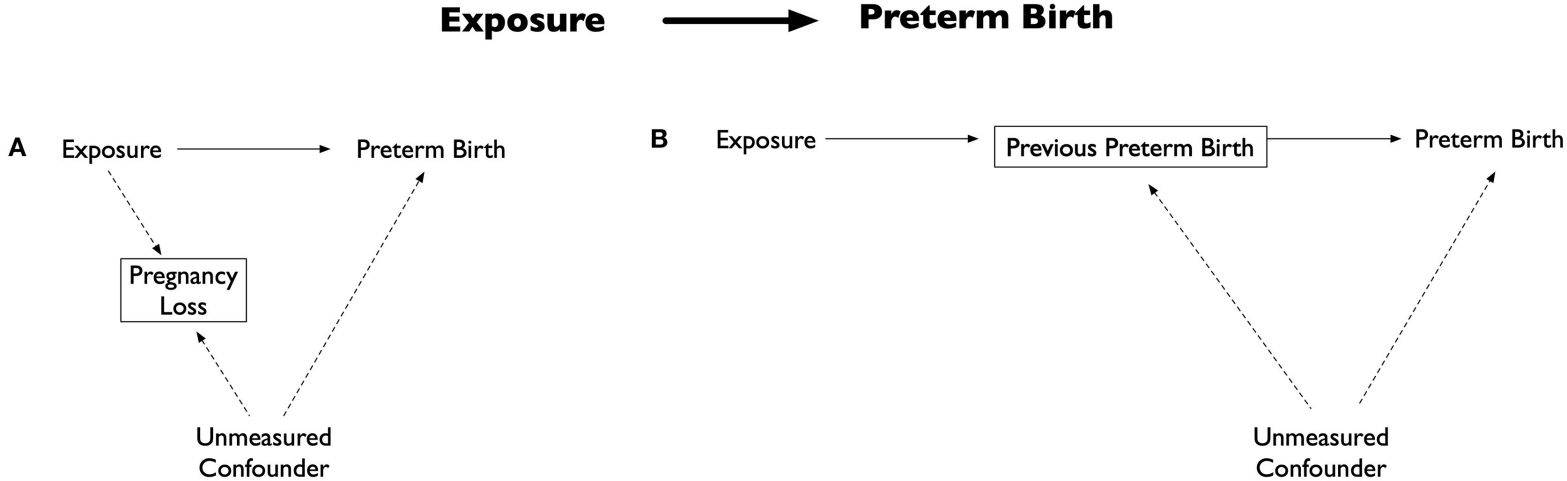
Directed acyclic graph illustrating selection bias examples. Adapted from: (19). **(A)** Potential bias by inclusion of only live births. The exposure is associated with outcome (preterm birth) and pregnancy loss, while the outcome and pregnancy loss are associated with an unmeasured confounder. Including only live births (essentially an adjustment of pregnancy loss) results in creation of a non-causal pathway between the exposure, unmeasured confounder and preterm birth. **(B)** Potential bias by adjusting for an intermediate variable. Commonly adjusted intermediate variable for the exposure and preterm birth is previous preterm birth. Adjustment of previous preterm birth results in creation of a non-causal pathway between the exposure, intermediate variable, unmeasured confounder and preterm birth.

Inappropriate analytical decisions can also introduce selection bias and impact the association between an exposure and PTB. For example, only including live born preterm infants, as often happens in such analyses, essentially adjusts for pregnancy loss. However, if an unmeasured confounder is associated with both pregnancy loss and preterm birth (Figure 2A), a non-causal pathway will be created potentially biasing estimates (19). Selection bias can also be induced through inappropriate variable treatment. Because PTB pathology is incompletely understood and unmeasured confounders cannot be adjusted for, it has become routine to treat independent risk factors as potential confounders, even though they could be intermediate variables. For example, previous PTB is often adjusted for because of its strong ties with future PTB risk (21). However, if unmeasured confounders are associated with both the intermediate variable and PTB, then this adjustment will create a non-causal pathway and potentially bias estimates (Figure 2B) (19).

##### Selection Bias: HIV/ART and Preterm Birth Examples

An analysis of birth outcomes including PTB was conducted in Malawi (22) among healthy HIV-infected and HIV-uninfected women. The authors hypothesized that ART use in pregnancy would eliminate previously observed differences in pregnancy outcomes by HIV status. Study enrolment occurred at delivery, with GA assessed postnatally (Ballard score). The overall PTB rate was 10%, with no differences observed by HIV status or ART status (initiation before or during pregnancy). The authors concluded that their results were consistent with their hypothesis that the efavirenz-based ART regimen eliminates differences previously attributed to HIV infection without introducing additional risk. However, these results differ from recent studies using similar ART regimens which found an increased risk of PTB by HIV/ART status (12, 14, 23). Although the authors dismissed the possibility of selection bias, there is a potential concern around the puerperium recruitment from a few facilities. This strategy likely excluded women with serious pregnancy complications, preterm infants, acutely ill neonates and/or neonatal deaths (24). The absence of very preterm (<32 weeks) and very low birth weight (<1,500g) infants in the study provides evidence for this. This unrepresentative study population could have led to bias toward the null resulting in the lack of difference observed by HIV and ART status (24).

Another example can be found in a systematic review conducted among HIV-infected women initiating ART before or during pregnancy, at a time when few women in LMICs conceived on ART (25). Preterm birth, reported in 10 studies, showed an increased risk among preconception initiators. It was hypothesized to be due to confounding by indication, with women on preconception ART likely to be at a more advanced HIV stage. The authors acknowledged the possibility of selection bias, emanating from the unequal opportunity for PTB between women starting late in pregnancy compared to those initiating ART preconception or early. Based on the selection concerns of this systematic review, a simulation trial was conducted to quantify the impact of the exclusion of women delivering pre-ART initiation, which shows that this exclusion lowered the risk of women included in studies investigating this association (26). In this simulation women were “recruited” preconception and randomized 1:1 to immediate ART (i.e., preconception) or delayed ART (i.e., during pregnancy). Gestational age at ART initiation and at delivery were based on previously collected Zambian data (27). Preterm birth rates were compared in Intention-to-treat (ITT) and naïve analyses. The ITT analysis compared all randomized women by ART initiation timing. While the naïve analysis, mimicking previous observational studies, compared all women randomized to immediate ART to a subset of women randomized to delayed ART (excluding those delivering pre-ART initiation). The ITT analysis showed no association with preconception initiation, while the naÏve analysis showed an increased risk with preconception initiation.

The selection biases highlighted here apply to all analyses investigating PTB and time-dependent exposures, because there will always be exclusion of women who deliver before experiencing or receiving the exposure later in pregnancy (26).

#### Information Bias

A certain amount of error is intrinsic in any measurement process. Information bias is the distortion of an association caused by the inaccurate measurement of key variables (28). This bias arises when information is either not accurately collected/measured or there is discord between study definitions and true definitions, leading to exposure or outcome misclassification (29). Gestational age (GA) is central in PTB studies, however it can only be estimated approximately because fertilization is a silent event. All methods of GA assessment (last menstrual period (LMP), symphysis fundal height measurement (SFH), and ultrasonography) are prone to varying degrees of error, primarily based on timing of assessment during pregnancy (30–32). Ultrasound is generally considered most reliable, however it is less accurate if carried out >24 weeks of pregnancy (32). SFH assessment is difficult early in pregnancy (33), and LMP reliability is limited by irregular menstrual cycles and inaccurate recall of dates (31). Postnatal assessments of the newborn by physical examination and neuromuscular assessment are even less precise (e.g., Ballard score) (34). Use of imprecise GA may introduce bias, with misclassification of preterm and term infants or preterm and small-for-gestational age infants.

Another major measurement issue relates to the timing of exposure. Human reproduction and development by nature is a highly timed and interrelated process (35). It spans across a spectrum of critical windows ranging from pre-pregnancy through to the early neonatal period (36), with any harmful exposures during these critical windows can increase PTB risk. Given this, quantification of the level and timing of exposures is essential for accurate classification of adverse outcomes. Some events are unobservable, so exposure quantification is further complicated by GA imprecision. Inappropriate treatment of exposures in analyses can also introduce bias. Although exposures are typically averaged over the entire pregnancy (by trimester or predefined lag period) in time-fixed analyses, this fails to capture the exposure windows of etiological importance (37). With time-varying exposures such as treatment in pregnancy (38), there is movement in and out of exposure states. Incorrectly treating the exposure as time-fixed ignores actual timing of treatment initiation, and person-time from the study start until delivery contributes wholly to the exposure classification (19, 39). This misclassification of the unexposed time before treatment initiation as exposed, considered as immortal time, can lead to biased estimates (40, 41).

##### Information Bias: HIV/ART and Preterm Birth Examples

Two studies investigating this association have shown the impact of gestational age measurement error. In South Africa, a birth outcomes study enrolled routine-care HIV-infected and HIV-uninfected women, with gestational age assessed antenatally using LMP, SFH and ultrasound (42). In a secondary analysis, PTB rates were compared based on assessment methods and their impact on the association with HIV/ART was examined (43). LMP-based GA was found to underestimate gestation relative to ultrasound-based gestational age, contributing to significant differences in preterm birth incidence estimates. An increased risk of PTB by HIV status was observed when GA was assessed using ultrasound, but associations were smaller and not statistically significant when GA assessment was by LMP and SFH. The discrepancy observed in findings between the GA assessment methods was considered to be due to random measurement error. This analysis highlights that the association between maternal ART use and preterm birth may be substantially influenced by GA assessment methods.

In Zambia, a study in routine care enrolled HIV-infected and HIV-uninfected women, with gestational age assessed using LMP and ultrasound (44); an increased incidence of PTB was observed when GA was assessed using LMP. The study also showed that LMP-based GA estimates were subject to under- and over-estimation depending on the timing of antenatal care presentation. Error in GA measurement is often thought to be random, as highlighted in the previous study, however these findings showed that LMP-based estimates of preterm among pregnancies may suffer from bias from systematic errors. This suggests that studies that rely on LMP alone are likely to falsely elevate the risk of preterm among groups of women who present later in pregnancy.

Subsequently, the authors of the previously discussed South African and Zambian cohorts conducted a combined analysis showing that ultrasound-based GA estimated PTB incidence was similar in both studies. However, substantial differences were observed in PTB incidence between the two cohorts, when using LMP-based GA: in the Zambian cohort (20.2%) PTB incidence was half of that in the South African cohort (39.7%) (43). In addition to recall issues, factors related to menstrual cycle variations and irregularities also contribute to LMP dating inaccuracies. The authors hypothesized that cohort differences could explain the differences seen with LMP-based estimates, in particular BMI profiles, with a significantly higher proportion of overweight/obese women in the South African cohort than in Zambia cohort (74 vs. 41%). Women with higher BMI tend to experience increased menstrual irregularity. This comparative analysis also highlights the need to improve understanding of maternal and fetal factors leading to biased GA estimates.

These examples of studies with unexpected or counterintuitive results highlight the importance of considering and addressing selection and measurement bias when designing and analyzing studies investigating the association between PTB and infections and/or their treatments.

## Conclusions

In this article, we briefly reflected on some important methodological considerations that should be regarded when designing studies or information systems for the monitoring or study of PTB, and the interpretation of findings. We described selection and information biases that can arise from inadequate study design, data collection, and analyses procedures, leading to inaccurate findings. The current approach for quantifying the estimates of PTB entails some necessary simplification, because of the limitations related to data availability. As part of achieving SDG 3, reducing PTB and its sequelae in LMICs is essential. We therefore advocate for the strengthening of antenatal care services to improve pregnancy outcomes and the quality of population data, particularly gestational age. The accuracy of this data is contingent on the availability of robust measurements tools such as ultrasound. However in LMICs availability is limited, therefore novel low-cost measurement tools need to be developed for improving gestational age assessment at the individual level. At the population level, leveraging the strengths of novel ML algorithms can strengthen the accuracy of PTB prediction and diagnosis (5). These efforts need to be coupled with improvements in birth registration systems, with the use of standardized definitions and classification by gestational age, and/or clinical presentation (spontaneous or medically-indicated PTB) in collection and reporting of data. Furthermore, continued capacity building of healthcare staff and researchers is critical, for the strengthening of data collection, management and analytic procedures.

We also need to optimize current study designs to study PTB and its risk factors more accurately, by taking into account the important methodological considerations described in this article. In particular, a better understanding and quantification of the error introduced when using commonly used, but error prone, assessment methods, so that PTB rates can be appropriately adjusted when necessary. Additionally, an nuanced understanding of data limitations is required, without which, results of global estimates or etiological research need to be interpreted with caution to avoid incorrect conclusions. New data analysis approaches should be explored as they may provide more efficient ways to use existing data to inform policy and practice.

Preterm birth remains a central public health issue, particularly in LMICs which bear the highest global burden. Despite this, the contribution of these populations to PTB data and research is limited. Further research efforts will require high quality epidemiologic and clinical data from these settings to inform development of context-specific interventions for PTB prevention and management.

## Data Availability Statement

The original contributions presented in the study are included in the article/supplementary material, further inquiries can be directed to the corresponding author/s.

## Author Contributions

TM conceptualized the perspective presented in this manuscript. TM and VR drafted the manuscript. M-LN and LM provided critical reviews to the manuscript. All authors contributed to the article and approved the submitted version.

## Funding

This work was funded by the Eunice Kennedy Shriver National Institute of Child Health and Human Development of the National Institutes of Health (No. R01HD080385) and L'Oreal-UNESCO for Women in Science South African National Young Talents Award.

## Author Disclaimer

The content is solely the responsibility of the authors and does not necessarily represent the official views of the National Institutes of Health or L'Oreal-Unesco for Women in Science.

## Conflict of Interest

The authors declare that the research was conducted in the absence of any commercial or financial relationships that could be construed as a potential conflict of interest.

## Publisher's Note

All claims expressed in this article are solely those of the authors and do not necessarily represent those of their affiliated organizations, or those of the publisher, the editors and the reviewers. Any product that may be evaluated in this article, or claim that may be made by its manufacturer, is not guaranteed or endorsed by the publisher.
